# Role of Gut Microbiota in Neuroendocrine Regulation of Carbohydrate and Lipid Metabolism via the Microbiota-Gut-Brain-Liver Axis

**DOI:** 10.3390/microorganisms8040527

**Published:** 2020-04-07

**Authors:** Shu-Zhi Wang, Yi-Jing Yu, Khosrow Adeli

**Affiliations:** 1 Institute of Pharmacy and Pharmacology, University of South China, Hengyang 421001, China; wangshuzhi727@126.com; 2 Hunan Province Cooperative Innovation Center for Molecular Target New Drug Study, University of South China, Hengyang 421001, China; 3 Molecular Medicine, Research Institute, The Hospital for Sick Children and Department of Physiology, University of Toronto, Toronto, ON M5G 1X8, Canada

**Keywords:** gut microbiota, carbohydrates, lipids, amino acids, central nervous system, enteric nervous system, gut peptides, bile acids, short-chain fatty acids, microbiota-gut-brain-liver axis

## Abstract

Gut microbiota play an important role in maintaining intestinal health and are involved in the metabolism of carbohydrates, lipids, and amino acids. Recent studies have shown that the central nervous system (CNS) and enteric nervous system (ENS) can interact with gut microbiota to regulate nutrient metabolism. The vagal nerve system communicates between the CNS and ENS to control gastrointestinal tract functions and feeding behavior. Vagal afferent neurons also express receptors for gut peptides that are secreted from enteroendocrine cells (EECs), such as cholecystokinin (CCK), ghrelin, leptin, peptide tyrosine tyrosine (PYY), glucagon-like peptide-1 (GLP-1), and 5-hydroxytryptamine (5-HT; serotonin). Gut microbiota can regulate levels of these gut peptides to influence the vagal afferent pathway and thus regulate intestinal metabolism via the microbiota-gut-brain axis. In addition, bile acids, short-chain fatty acids (SCFAs), trimethylamine-N-oxide (TMAO), and Immunoglobulin A (IgA) can also exert metabolic control through the microbiota-gut-liver axis. This review is mainly focused on the role of gut microbiota in neuroendocrine regulation of nutrient metabolism via the microbiota-gut-brain-liver axis.

## 1. Introduction

It has been reported that gut microbiota regulate the metabolism of carbohydrates, lipids, and amino acids, which play an important role in human health and metabolic diseases [1]. The gut microbiota is estimated to contain trillions of microorganisms, with more than 100 bacterial species, which are mainly divided into six phylums: *Firmicutes*, *Bacteroidetes*, *Proteobacteria*, *Actinobacteria*, *Verrucomicrobia* and *Fusobacteria*. Among them, the *Firmicutes* (such as *Clostridium*, *Enterococcus*, *Lactobacillus*, and *Ruminococcus*) represent up to 60% of the gut microbiota, and the *Bacteroidetes* (such as *Bacteroides* and *Prevotella*) represent up to 15%, while other species own a smaller proportion [2,3,4]. On one hand, gut microbiota are changing with human development and are influenced by dietary habits, health status, drugs, and so on [5,6,7]. On the other hand, gut microbiota can affect carbohydrate, lipid, and amino acid metabolism, and thus influence several metabolic diseases, such as obesity, type 2 diabetes, dyslipidemia, non-alcoholic fatty liver disease (NAFLD), gout, vitamin deficiency, and atherosclerosis [1,8,9,10]. At the same time, gut microbiota regulate metabolic status and host health by producing short-chain fatty acids (SCFAs) via carbohydrate fermentation. The major SCFAs are formate, acetate, propionate, and butyrate, which are related to maintaining intestinal epithelium and permeability [11]. Further, SCFAs regulate glucose and lipid metabolism as well as immune and inflammatory responses [12,13]. Hence, gut microbiota also plays an important role in immune systems, inflammation, and cancer prevention of the host [14,15].

The enteric nervous system (ENS) is reported to be involved in intestinal metabolic regulation, and enteric neurons and intestinal neurotransmitters play an important role in ENS regulation [16,17,18]. The gut contains full ENS reflex circuits, such as motor neurons, interneurons, and sensory neurons, and these neurons transfer information between the ENS and central nervous system (CNS). The vagal nerve pathway communicates between the CNS and ENS, which has remarkable impact on regulating gastrointestinal tract functions and feeding behavior [19,20]. Thus, the vagal nerve system is also involved in intestinal metabolic regulation through the gut-brain axis. Vagal afferent neurons express receptors for gut peptides, such as cholecystokinin (CCK), ghrelin, leptin, peptide tyrosine tyrosine (PYY), glucagon-like peptide-1 (GLP-1), 5-hydroxytryptamine (5-HT) and so on, which are secreted from enteroendocrine cells (EECs) [20,21,22]. When vagal afferent neurons sense these types of gut peptides, the corresponding gut information will transfer to the CNS and exert various reactions. At the same time, gut microbiota can regulate these gut peptides, such as CCK, ghrelin, leptin, PYY, GLP-1, 5-HT levels to influence vagal afferent pathway, and then regulated intestinal metabolic metabolism via the microbiota-gut-brain axis [23,24,25].

The importance of the gut-brain axis in human health and disease has been known for a long period. However, it has only been recently recognized that the intestinal microbiota are key regulators of crosstalk between the brain and the gastrointestinal tract to maintain metabolic homeostasis, which is called the microbiota-gut-brain axis [26,27,28]. The bidirectional communication that occurs for the microbiota-gut-brain axis includes “bottom-up” (from gut microbiota to brain) and “top-down” (from brain to gut microbiota) [29,30]. For example, gut microbiota can regulate levels of gut peptides to influence the vagal afferent pathway and regulate intestinal metabolism via the microbiota-gut-brain axis [23,24,25].

Several studies have also confirmed that there are various links between the gut and the liver, and that gut microbiota are also involved in intestinal metabolism through the microbiota-gut-liver axis [31,32]. Therefore, this article mainly summarizes the role of the gut microbiota in neuroendocrine regulation of carbohydrates, lipids, and amino acids via the microbiota-gut-brain-liver axis.

## 2. Nutrient Metabolism (Carbohydrate, Lipid, Amino Acid) and Their Interaction with Gut Microbiota

### 2.1. Carbohydrate Interaction with Gut Microbiota

Carbohydrates have become one of the most studied dietary components associated with gut microbiota modifications. Alterations in dietary carbohydrates have important effects on the composition and function of gut microbiota [3,33]. In one dietary intervention study, a low-fat, high-carbohydrate diet for 24 weeks increased both faecal *Bacteroides* and *Bifidobacteria*, which have both been related to improved body energy regulation and reduced risk factors for obesity and metabolic syndrome [34]. In contrast, several studies have shown that reducing carbohydrate intake resulted in a remarkable decrease in *Bifidobacteria*, as well as some *Clostridium* subgroups (*Roseburia* and *Eubacterium rectale*), which reduced butyrate levels in stool [35,36].

Likewise, different types of dietary carbohydrates can also induce remarkable alterations in gut microbiota. For example, a recent study revealed that a high-fat, high-sucrose diet reduced relative abundance of *Bacteroides*/*Prevotella* spp. and *Clostridium* cluster IV, and also increased the relative abundance of *Enterobacteriaceae*, in comparison to a chow-fed diet [37]. Alterations in the relative abundance of other gut microbiota were also observed at different time points in Sprague-Dawley rats fed a high-fat, high-sucrose diet [37]. Another study suggests that different forms of fructose consumption can change gut microbiota, intestinal mucosa integrity, and liver homeostasis [38]. The authors found that *L-Ruminococcus*, *Coprococcus* and *R-Ruminococcus* were increased, and the *Firmicutes*/*Bacteroidetes* ratio was decreased, in mice fed with fructose in comparison to a standard diet [38]. Llewellyn et al. reported that different types of carbohydrates altered gut microbiota density and intestinal permeability, affecting colitis severity in mice [39]. The authors categorized dietary carbohydrates into those that are digestible for the host, including sucrose, fructose, glucose, maltodextrin, and corn starch, and those that are mainly indigestible for the host (i.e., fiber), including cellulose, methylcellulose, psyllium, pectin, inulin, flaxseed, marshmallow root, potato starch, and slippery elm, and then each diet was fed to the mice. Of the nine fiber sources, psyllium, pectin, and cellulose fiber reduced the severity of colitis in mice, while methylcellulose increased the severity of colitis in mice. Interestingly, psyllium fiber reduced the severity of colitis through microbiota-dependent mechanisms. The authors also reported that a high-psyllium diet (HPSY) markedly decreased microbiota density compared to a high-cellulose diet (HCEL), as the diversity of fecal microbiota was much greater in mice fed with HPSY compared to mice fed with HCEL [39]. 

While gut microbiota can be modified by dietary carbohydrates, they can also affect carbohydrates by playing a role in their metabolism. Humans have limited ability to degrade complex polysaccharides and non-digestible carbohydrates, such as resistant starch, non-digestible polysaccharides, and oligosaccharides and plant fibers [40]. These non-digestible substrates enter the colon and are then processed by the colon microbiota [41]. The colonic microbiota produce various hydrolytic enzymes for the degradation of these substances. The complex carbohydrates are converted into polysaccharides through primary degradation, which are then converted into oligosaccharides [33]. Therefore, gut microbiota use these indigestible carbohydrates as their main energy sources [42]. *Bacteroides* species have been reported as the main carbohydrate-degrading bacteria in the gut and help to degrade complex pectin [43,44]. It was reported that *Bacteroides* have a variety of glycans and glycosidases that can utilize polysaccharides, while *Bifidobacterium* utilize carbohydrates of low molecular weight [45,46]. In another study, Tingirikari summarized the mechanisms that involved in the utilization of carbohydrates by *Bacteroides* and *Firmicutes*. This article also showed that polysaccharides were degraded into oligosaccharides along with synthesis of SCFA by *Bacteroides* and *Firmicutes* in different regions of the colon [47].

SCFAs produced by microbiota via carbohydrate fermentation may have beneficial effects on the host. SCFAs signal through the CNS and several G protein–coupled receptors (GPCRs) to regulate a series of physiological processes, including carbohydrate and lipid metabolism, energy homeostasis, and inflammatory signals inhibition [48,49]. However, even though SCFAs produced by gut microbiota have displayed protective effects in many studies, studies with obese humans and ob/ob genetically obese mice show increased caecum concentrations of SCFAs and less energy remaining in the faeces, indicating that SCFAs may contribute to enhanced energy harvest in obese status [50].

In recent years, there has been an upsurge in the research of probiotics for modulation of gut microbiota and related improvements in human health [51,52]. Nowadays, probiotics have become commercial microbial dietary supplements that positively affect the host via their effects in the intestinal tract. The two major genera of gram-positive bacteria, *Lactobacillus* and *Bifidobacterium*, are widely used as probiotics. However, other species, such as *Bacillus cereus*, *Enterococcus faecalis*, *Escherichia coli Nissle*, *Streptococcus thermophilus* and *Saccharomyces boulardii*, have also been classified as probiotic [53]. Probiotics have been reported to show beneficial effects on human health, such as reducing and preventing diarrhea of different sources, improving the intestinal microbial balance by antimicrobial activity, relieving lactose intolerance symptoms, preventing allergic diseases, stimulating immune system efficacy, and anti-tumor activities. Further, probiotics can improve obesity, insulin-resistance syndrome, type 2 diabetes and NAFLD [54,55,56].

A variety of dietary fibers and prebiotics can also improve microbiota diversity and richness [57]. Prebiotics, defined by Gibson and Roberfroid, are non-digestible food ingredients that can be beneficial via selectively stimulating the growth and/or activity of one or a limited amount of bacterial species already resident in the gastrointestinal tract, thereby improving host health [58]. The most commonly used prebiotics include oligosaccharides, inulin, fructooligosaccharides (FOS), and isomalto-oligosaccharides. These prebiotics provide additional support for probiotics [59]. An appropriate combination of probiotics and prebiotics are called synbiotics [60], which may exert superior effects on enhancing health functions.

Since carbohydrate metabolism is directly related to obesity and diabetes, the gut microbiota also play an important role in these diseases. Ley et al. found that *ob*/*ob* mice presented a 50% reduction in the abundance of *Bacteroidetes*, and a proportional increase in *Firmicutes*, as compared to lean mice. The authors also pointed out that an increased ratio of *Firmicutes* to *Bacteroidetes* may help promote adiposity in *ob*/*ob* mice [61]. Another study demonstrated that alteration in gut microbiota increased the capacity to harvest energy from the diet in *ob*/*ob* mice [50]. In humans, several studies have shown that the ratio of *Firmicutes* to *Bacteroidetes* significantly affects body mass index (BMI) and childhood obesity [62].

Similar to obesity, diabetes is also correlated with gut microbiota alteration. A recent study showed that altered gut microbiota composition (i.e., dysbiosis) is markedly correlated with insulin dysfunction and type 1 diabetes [63]. Murri et al. performed a case-control study about fecal bacteria composition in 16 children with type 1 diabetes and 16 healthy children. In the children with type 1 diabetes, *Actinobacteria*, *Firmicutes* and the *Firmicutes* to *Bacteroidetes* ratio were decreased compared to the healthy children, while *Bacteroidetes* increased. Further, there was a remarkable increase in the number of *Clostridium, Bacteroides*, and *Veillonella*, and a significant decrease in the number of *Lactobacillus*, *Bifidobacterium*, *Blautia coccoides*/*Eubacterium rectale* group and *Prevotella*, in the children with type 1 diabetes. The authors also found that the gut microbiota alterations correlated with plasma glucose level in the children with type 1 diabetes. The results showed that numbers of *Lactobacillus* and *Bifidobacterium* were negatively associated with plasma glucose levels, while numbers of *Clostridium*, *Bacteroides*, and *Veillonella* were positively associated with plasma glucose levels in the children with type 1 diabetes [64]. In another study, Qin et al. performed an analysis on gut microbiota content in patients with type 2 diabetes and found that they had moderate gut microbiota dysbiosis. The abundance of some butyrate-producing bacteria, such as *Clostridiales* sp. SS3/4, *Eubacterium rectale*, *Faecalibacterium prausnitzii*, *Roseburia intestinalis*, and *Roseburia inulinivorans*, were decreased in patients with type 2 diabetes compared to healthy individuals. In addition, some opportunistic pathogens, such as *Bacteroides caccae*, *Clostridium hathewayi*, *Clostridium ramosum*, *Clostridium symbiosum*, *Eggerthella lenta*, and *Escherichia coli*, were increased in patients with type 2 diabetes [65]. Therefore, targeting gut microbiota may have therapeutic potential for patients with obesity or type 1 and type 2 diabetes.

### 2.2. Lipid Interaction with Gut Microbiota

The ratio and source of dietary fat are important to intestinal microbiota modification [66]. Alpha diversity is a measurement of how many taxa exist, as well as the distribution of taxa, which trended toward significance between low and high saturated fat levels (two-way analysis of variance (ANOVA), *F* = 2.97, *P* = 0.088) in recent research [67]. The authors also reported that gut microbiota taxon abundance was affected by saturated fat levels. They indicated that there were 151 differentially abundant operational taxonomic units (OTUs) between low and high saturated fat level diets, of which 57 were described at the genus level [67]. Otherwise, compared to low-fat diets, high-fat diets have frequently shown to increase the abundance of intestinal microbiota [68,69]. 

The effects of plant- and animal-based fat sources on the gut microbiota are notably different. Muralidharan et al. reported that intestinal microbiota was differentially modified by various kinds of fats from nuts, peanuts, almonds, corn oil, coconut oil, olive oil, sesame oil, soybean oil, and the Mediterranean diet [70]. This study suggested that nuts and other plant-based sources of fats increased genus *Bifidobacterium*, *Roseburia*, and *Faecilibacterium*, which have been related to positive metabolic effects [70]. Other research investigated the effects of different lipid diets (low fat, milk fat, olive oil or corn oil) on the gut microbiota. This study indicated that milk fat and corn oil diets led to increased alpha diversity of gut microbiota in mice [68]. In addition, observed species richness and Chao1 were increased with exposure to milk fat and corn oil, while the richness caused by the olive oil diet was similar to the low-fat chow diet [68]. 

Likewise, the effects of saturated and unsaturated fats on the intestinal microbiota are markedly different. Patrone et al. reported that two different diets enriched with coconut oil (high in saturated fat) or soy oil (high in polyunsaturated fat) showed different diversity and metabolic capacity of the cecum bacterial community in C57BL/6 N mice [71]. This research demonstrated that in comparison with soy oil (high in polyunsaturated fat) diet-fed mice, coconut oil (high in saturated fat) diet-fed mice presented an increased relative abundance of *Allobaculum*, *Anaerofustis*, F16, *Lactobacillus reuteri* and *Deltaproteobacteria*, along with a decreased relative abundance of *Akkermansia muciniphila* in the cecum microbiota. Another study indicated that high saturated fat diet increased the ratio of gram-negative intestinal microbiota, and then increased endotoxemia [72]. Meanwhile, Mani et al. investigated the effects of different dietary oil treatments on ex vivo endotoxin transport in pig ileum tissue, and they found cod liver oil and fish oil (unsaturated fat) deceased gut permeability, while coconut oil (saturated fat) increased gut permeability. This research suggests that enteroendotoxin transport may be regulated by lipid raft-mediated mechanisms, and saturated fat may stabilize the lipid rafts allowing for greater endotoxin transport, which indicates that saturated fat may lead to metabolic endotoxemia via increasing intestinal permeability and changing the gut microbiota composition [73].

While gut microbiota can be modified by dietary fat, they can also affect lipids by playing a role in their metabolism [74]. Several studies have reported that gut microbiota modulate lipid metabolic abilities of the host through regulating absorption, storage, and energy harvest from the diet [75,76]. Backhed et al. demonstrated that when adult germ-free (GF) C57BL/6 mice were treated with a normal microbiota harvested from the cecum of conventionally raised animals they experienced a 60% increase in body fat content and insulin resistance over 14 days in comparison with GF mice, despite lower food intake. This study also observed that microbiota promoted de novo hepatic lipogenesis together with induced energy harvest from the diet and energy storage in the host [75]. In another study, Fu et al. studied 893 subjects from the Life-Lines-DEEP population cohort [77]. The authors identified 34 intestinal bacterial taxa which were correlated with BMI and blood lipids. They found gut microbiota was related to 4.5% of the variation in BMI, 6.0% in triglycerides (TG) and 4% in high-density lipoproteins (HDL), which was independent of age, sex, and genetic risk factors [77]. Otherwise, gut microbiota were also reported to regulate metabolic disorders such as obesity, type 2 diabetes, NAFLD and atherosclerosis [76,78].

SCFAs and bile acids have been reported to act as important regulators involved in the mechanisms that gut microbiota affected lipid metabolism [79,80,81]. A recent research study characterized the fecal microbiota characteristics, SCFAs, blood lipids and bile acids profile in hypercholesterolemic subjects [81]. The authors indicated that *Anaeroplasma* and *Haemophilus* were negatively correlated to cholesterol (CHOL) and TG related biomarkers and positively correlated to HDL size, while *Odoribacter* presented the opposite effect. The authors also found that hypercholesterolemic subjects showed higher abundance of isobutyric and isovaleric acid in fecal SCFAs profile compared to normal subjects. The isobutyric acid was positively related to *Odoribacter* and an unfavourable lipid profile. Other studies have shown that gut microbiota can also regulate the metabolism of bile acids that are ligands for nuclear receptor Farnesoid X receptor (FXR) and the membrane receptor GPCR 5 (TGR5), which are involved in the regulation of glucose, lipids, and energy balance [82,83]. Pathak et al. demonstrated that gut microbiota played an important role in bile acids metabolism and FXR/TGR5/GLP-1 signaling, which induced adipose tissue browning and improved hepatic glucose and insulin sensitivity [82]. Another study reported that gut microbiota increased the diversity of bile acids and then regulated CHOL- and lipid-related pathways in the distal ileum in males and females [84].

### 2.3. Amino Acid Interaction with Gut Microbiota

Amino acids produced by food or the host can provide nutrients for gut microbiota and support their protein synthesis [85]. Alterations in dietary protein may lead to changes in composition and function of gut microbiota. For example, a high-protein diet can increase the pH of small intestine, increasing the numbers of *Escherichia coli* and reducing the numbers of *Akkermansia muciniphila*, *Bifidobacterium*, *Prevotella*, *Ruminococcus bromii*, and *Roseburia/Eubacterium rectale* in comparison to the normal-protein diet in a rat model [86]. Another study, reported by Li et al., investigated the difference in gut microbiota between a high-protein, low-carbohydrate diet (HPLC) and a low-protein, high-carbohydrate diet (LPHC) in dogs. The results demonstrated that a LPHC diet was beneficial for the growth of *Bacteroides uniformis* and *Clostridium butyricum*. However, the HPLC diet increased the abundances of *Clostridium hiranonis*, *Clostridium perfringens*, and *Ruminococcus gnavus*, together with improved microbial gene networks that related to weight maintenance [87].

Different types of dietary protein also induced remarkable alteration of gut microbiota. An et al. investigated the differences among cecum microbiota in rats that were fed with milk-casein, soy-protein, and fish-meal [88]. This study demonstrated that rats fed with soy-protein presented greater diversity of microbiota. Meanwhile, the proportion of *Bifidobacterium* was higher in the milk-casein diet group as compared to other diet groups, which proved the enhancement effect of casein on *Bifidobacterium*. Interestingly, *Lachnospiraceae*, and *Parasutterella excrementihominis* were only present in rats fed with fish-meal diet [88]. Thus, the authors showed that different dietary proteins affected the intestinal environment.

Meanwhile, amino acid composition changes in diet can play an important role in gut microbiota alteration, which may affect the species and metabolism of the amino acid-fermenting bacteria and influence the metabolism of the host. For example, branched-chain amino acids, such as leucine, isoleucine, and valine, were reported to promote intestinal development in piglets [89,90]. Yang et al. found that branched-chain amino acid-enriched mixture supplementation in the diet altered the gut microbiota structure in BALB/C mice. Meanwhile, these mice displayed an increased abundance of the *Akkermansia* and *Bifidobacterium*, along with a decreased ratio of *Enterobacteriaceae*. This study also proved that branched-chain amino acid-enriched mixture supplementation was beneficial for the host health [91]. Agus et al. summarized that the essential amino acid tryptophan has three metabolic pathways to induce serotonin, kynurenine, and indole derivatives, which affect gut microbiota via gut-brain signaling [92]. Another research study showed that a lysine-restricted diet altered the species and abundances of gut microbiota and mediated amino acid metabolism, which also changed levels of hormones, such as leptin, CCK, and ghrelin in piglets [93].

While gut microbiota can be altered by amino acids, they can also act to help maintain host amino acid homeostasis by promoting the digestion and absorption of amino acids and synthesizing several amino acids that are necessary for the host [94]. Dai et al. summarized the main amino acid-fermenting bacteria in the gastrointestinal tract of humans and animals, which included *Clostridium*, *Fusobacterium*, *Peptostreptococcus*, *Veillonella*, *Megasphaera elsdenii* and *Selenomonas ruminantium* [95]. Among them, bacteria of the *Clostridium* genus play an important role in the fermentation of lysine and proline, while bacteria of the *Peptostreptococcus* genus are related to glutamate and tryptophan metabolism. Amino acids are mainly absorbed in the small intestine and are mediated by several kinds of bacteria genus, such as *Prevotella ruminicola*, *Butyrivibrio fibrisolvens*, *Megasphaera elsdenii*, *Selenomonas rum inantium*, and *Streptococcus bovis* [95]. In addition, gut microbiota also contribute to *de novo* biosynthesis of amino acids. For example, Deguchi et al. reported that intestinal bacteria helped incorporate ^15^N from ammonium chloride or urea into lysine in pigs [96]. Another research study also proved that gut microbiota of pigs promoted the incorporation of ^15^N from ^15^NH_4_Cl together with ^14^C from ^14^C-polyglucose in the diet into essential amino acids that are required by the host, such as lysine, valine, isoleucine, leucine, and phenylalanine [97]. In another aspect, Mardinoglu et al. [98] showed that intestinal microbiota regulated amino acid metabolism of the host and then affected glutathione (GSH) metabolism. This study found that, compared to GF mice, conventionally raised (CONV-R) mice presented lower levels of glycine and serine in the portal vein because small intestinal microbiota may consume glycine and other amino acids to maintain its growth and survival. The CONV-R mice also showed lower levels of *de novo* GSH synthesis due to glycine reduction. Meanwhile, Nnt expression was found to be increased in the liver in CONV-R mice, which is related to insulin sensitivity [98].

## 3. Interaction between Gut Microbiota and Enteric Neurons and Vagal Signaling

The ENS contains various kinds of enteric neurons, such as motor neurons to the muscle, intrinsic arterioles and epithelium, interneurons, intrinsic primary afferent neurons, secretomotor and vasomotor neurons, and so on [99]. In fact, 14 functionally-defined neuron types have been found in the small intestine nervous system of guinea pigs. These enteric neurons were shown to contribute to the effects of the ENS on movement, blood flow, sensation, absorption, secretion, and communication. In humans, the total number of enteric neurons is 400–600 million. Enteric and exogenous neurons connect to the gastrointestinal tract through various transmitters, which include primary transmitters, secondary transmitters and other neurochemical modulators [16]. The primary transmitters contain acetylcholine (ACh), nitric oxide (NO), adenosine triphosphate (ATP), noradrenaline and gastrin releasing peptide (GRP), and secondary transmitters mainly contain vasoactive intestinal peptide (VIP), 5-HT, tachykinin, and pituitary adenylyl-cyclase activating peptide (PACAP), while other neurochemical modulators contain calretinin, enkephalin, somatostatin, neuropeptide Y (NPY), opioid peptides, and CCK [16].

Previous studies have reported that gut microbiota affect intestinal movement, metabolism, immune responses, and behaviors via mediating enteric neurons [100,101]. Previous studies have demonstrated that the jejunum and ileum of GF mice showed a smaller number of neuronal cell bodies per ganglion and decreased nerve density in comparison to specific pathogen-free (SPF) mice [102]. Anitha et al. showed that a reduction in neurons and gastrointestinal motility retardation presented in GF mice as compared to wide-type mice, indicating that gut microbiota play an important role in neurons survival and gastrointestinal motility [103]. Gut microbiota can affect primary and secondary bile acid metabolism and mediate TGR5 and GLP-1 secretion, which control enteric neurons and gut motility. In addition, gut microbiota can influence enteric neurons and gut motility through regulating SCFA and 5-HT in the gut. Toll-like-receptor (TLR) signaling, such as TLR2 and TLR4, expressed by enteric neurons can also contribute to gut motility [104]. In contrast, TLR2^−/−^ mice showed enteric neuron alterations and abnormal gut motility, and wild-type mice also exhibited these results, along with depletion of gut microbiota [105]. This study confirmed that TLR2 expressed by enteric neurons, along with gut microbiota, regulated ENS structure and intestinal function [105]. On another note, gut microbiota can also play an important role in enteric neurons function through regulating other intestinal hormones and endocrine peptides synthesis, such as NPY, PYY, CCK, pancreatic polypeptide, corticotropin-releasing factor, ghrelin, and so on [106].

The vagal nerve pathway communicates between the CNS and ENS, which is reported to have remarkable impact on regulating gastrointestinal tract functions and feeding behavior. The vagal nerve is the main component of the parasympathetic nervous system and consists of 80% afferent fibers and 20% efferent fibers [29]. Vagal afferent nerve endings are widely distributed in the stomach and proximal small intestine mucosal layers. Vagal afferent neurons express receptors for gut peptides, such as CCK, ghrelin, leptin, PYY, GLP-1, 5-HT, and so on, which are secreted from gastrointestinal enterochromaffin cells (ECCs) and EECs [21]. When vagal afferent neurons sense these types of gut peptides, the related gut information will transfer to the CNS, which then exerts various responses.

Gut microbiota cannot contact vagal afferent fibers directly [29]. However, as we previously discussed, gut microbiota can regulate gut peptides to influence vagal afferent pathway. For example, vagal afferent neurons express CCK type 1 receptors for CCK. Zhang et al. showed CCK level increased together with GLP-1 and PYY levels in the ileum, colon, and cecum in moderate fructose malabsorption (ketohexokinase mutant) mice who received 20% fructose diet, which may be related to a gut microbiota dependent process [107]. In another study, plasma levels of CCK, ghrelin, PYY, GLP-1, and GLP-2 were found to be different between severely obese and healthy normal-weight patients, which was associated with differences in microbiota composition [108]. In other studies, gut microbiota have been shown to affect 5-HT release from EECs, which activates 5-HT3 and 5-HT2 receptors expressed on vagal afferent fibers [109,110]. Further, SCFAs including acetate, propionate, and butyrate, which are produced by gut microbiota, have been shown to activate the vagal afferent pathway and suppress food intake [111]. In addition, lipopolysaccharides (LPS) produced by gram-negative bacteria in the intestine can also influence the vagal afferent pathway and induce inflammatory responses and obesity. Specifically, the TLR4 receptor expressed on vagal afferent fibers can sense LPS and then transfer the signal to the brain [112,113]. In conclusion, gut microbiota regulate gut motility, gastrointestinal secretion, and food intake through their indirect effect on the vagal afferent pathway.

## 4. Microbiota-Gut-Brain Axis

The importance of the gut-brain axis in human health and diseases has been appreciated for a long time. However, it has only been recognized in the past decade that gut microbiota serve as key regulators in the crosstalk between the brain and gastrointestinal tract to maintain homeostasis, creating the new term “microbiota-gut-brain axis” [26,114]. The role of microbiota in gut-brain interaction was first uncovered in the study of GF mice, as GF mice exhibited exaggerated hypothalamic–pituitary–adrenal (HPA) stress, when compared to control specific pathogen-free mice [115]. Reconstitution of *Bifidobacterium infantis* reversed the exaggerated HPA stress response in GF mice [115]. Since then, a series of observations, based on microbiota perturbation models (GF or antibiotic-treated animals), probiotic, or bioactive treatment models and fecal transplantation in animal models or human trials, indicated that gut microbiota are not only essential for maintaining normal neurophysiology and behaviors [116], but also influence the pathogenesis of various diseases, including neurologic disorders, gastrointestinal diseases, and metabolic disorders [117,118].

The gut microbiota can communicate with the CNS via five different communication routes: (1) the neuroanatomical pathway; (2) the neuroendocrine-HPA axis pathway; (3) the immune system; (4) the gut microbiota metabolism pathway; and (5) the intestinal mucosal barrier and blood-brain barrier (BBB) [119]. The bidirectional communications occur for the microbiota-gut-brain axis: “bottom-up” (from gut microbiota to brain) and “top-down” (from brain to gut microbiota) [29,30].

### 4.1. Mechanisms of Bottom-Up Communication

The bottom-up communication mainly occurs through neuroendocrine and neuroimmune systems, involving the neuroendocrine EECs and ECCs, intestinal mucosal barrier, and BBB [29,30]. The metabolites of gut microbiota, such as SCFAs, tryptophan metabolites and secondary bile acids, are key players mediating this bottom-up communication [29,30].

The neuroendocrine cells, which can secrete various kinds of hormones or neuropeptides are part of the widely distributed neuroendocrine regulatory system [120]. The HPA axis represents the major neuroendocrine system, which mounts an adequate response to the stressor [121]. Environmental stress, as well as elevated systemic pro-inflammatory cytokines, can activate this system; the corticotropin-releasing factor (CRF) secreted from the hypothalamus stimulates adrenocorticotropic hormone (ACTH) secretion from pituitary gland and ACTH, in turn, leads to the release of cortisol from the adrenal glands [121]. EECs are located in the gastrointestinal mucosa layer and represent only 1% of epithelial cells in the gut. However, EECs confer the gut as the biggest endocrine organ of human body [122], since they secrete more than 20 peptides and hormones [123]. In the bottom-up interaction of the microbiota-gut-brain axis, EECs express various receptors that respond to gut microbiota metabolites, acting as the sensors for gut microbiota metabolites. For instance, EECs express several GPCRs, which can act as receptors for SCFAs derived from gut microbiota [124,125]. SCFAs are produced by bacterial fermentation of host non-digestible carbohydrates, which serve as the important host’s fuel. Many studies have suggested that SCFAs can regulate the release of gut-derived satiety hormones from endocrine cells, in particular GLP-1 and PYY [126,127]. In addition to SCFAs, bile acids are another group of well-studied microbiota metabolites. Primary bile acids are produced in the liver, and then released into the intestine, where they are converted into secondary bile acids. Since secondary bile acids are mainly generated by specific gut bacterial enzymes, gut microbiota can control the homeostasis of bile acids, thus impacting various host pathophysiological processes. EECs express the bile acid receptors, such as nuclear receptor FXR and cell membrane receptor TGR5 [128,129,130]. A few studies have indicated that activation of TGR5 or FXR in EECs resulted in secretion of GLP-1 from these cells [82,130,131]. Furthermore, EECs respond to bacterial LPS via TLRs [132].

ECCs are another type of intestinal endocrine cell, which secrete >90% of the whole-body serotonin (also known as 5-HT). Brain 5-HT has been linked to mood, social behaviors, sleep, depression, appetite, sex, and temperature control [133]. The role of gut 5-HT has been partially uncovered recently [134], and it includes regulating gastrointestinal motility and secretion, nausea, and visceral hypersensitivity [135]. A few studies have revealed gut microbiota can affect the level of gut serotonin. For instance, GF mice exhibited approximately 3-fold lower levels of plasma serotonin compared to conventional SPF mice [136]. Furthermore, gut bacteria depletion by antibiotic treatment resulted in significantly lower 5-HT and 5-HT-positive staining cells in colon tissues [137]. In addition, microbiota from humanized and conventionally raised mice significantly increased the expression of serotonergic genes, including tryptophan hydroxylase 1 (rate-limiting enzyme for mucosal 5-HT synthesis) and chromogranin A (involved in neuroendocrine secretion) [138]. It has also been demonstrated that SCFAs (but not LPS), derived from gut microbiota, induced the production of serotonin in human ECCs [138]. Yano et al. showed that gut microbiota can regulate the 5-HT levels in plasma and colonic tissues, and their metabolites, such as SCFAs and bile acids, can directly mediate 5-HT secretion in ECCs [139].

Besides the interaction with ECCs, it has been shown that microbiota, or their metabolites, including SCFAs and LPS, can directly activate the vagus nerve [140,141]. For instance, oral administration of *Campylobacter jejuni* activated the vagal neurons in mice as evidenced by increased c-Fos expression in these neuronal cells in the absence of increased levels of pro-inflammatory cytokines [142]. A high level of indole, a metabolite produced by gut microbiota from tryptophan, resulted in vagus nerve activation in GF rats [143]. Moreover, polysaccharide A, produced by certain gut bacteria, can directly activate the intestinal afferent neurons [144].

In addition to metabolites, microbiota can produce a wide range of neurotransmitters, such as gamma-aminobutyric acid, serotonin, acetylcholine, histamine, norepinephrine, and dopamine [145]. Accumulating evidence suggests that these neurotransmitters produced by gut bacteria impact host physiology, and this topic has been well discussed in other reviews [28,145]. Additionally, microbiota have been indicated to alter the production of several neurotransmitters, including gasotransmitters, neuropeptides, and endocannabinoids [145].

The intestinal mucosal barrier and BBB are the two major natural barriers that regulate the signaling exchange within the microbiota-gut-brain axis. The structure and function of the intestinal mucosal barrier can be influenced by alterations in gut microbiota, host stress, and inflammation. It has been revealed that an impaired intestinal barrier and altered microbiota have a great impact on the pathogenesis of many diseases, including several inflammatory diseases, metabolic disorders, and mental diseases [146]. However, it is not well understood how gut microbiota influence the function and structure of the intestinal barrier under physiological conditions. Recently, Hayes et al. has shown that the colonization of gut commensal bacteria in mice promoted the structural and functional adaptations of the intestinal barrier, which is essential for the maintenance of intestinal homeostasis [147]. The BBB serves as a message and nutrient exchange gate between the blood circulation and brain parenchyma. Gut bacteria produce several metabolites that can cross the BBB [148]. In addition, Braniste et al. suggested that gut microbiota can regulate the BBB integrity in both fetal and adult brains of mice, as the authors observed disorganized tight junction of the BBB in GF mice and an improvement in the BBB integrity following microbiota colonization [149]. Furthermore, gut microbiota metabolites, including SCFAs, have been shown to improve the integrity of both the BBB and intestinal barriers [149,150].

### 4.2. Mechanisms of Top-Down Communication

The top-down communication between the brain and gut microbiota can occur in several ways, including neuroanatomical pathway, regulation of intestinal barrier and release of neurotransmitters (e.g., 5-HT and catecholamines) [151,152,153]. 

The autonomic nervous system (ANS) acts as a control system and regulates various body functions, such as heartbeat, breathing, urination, and digestion. The ANS is comprised of the sympathetic, parasympathetic and ENS branches. Direct or indirect enteric neuron system-microbiota interactions can occur as a result of ANS activity. Regarding gastrointestinal function, the ANS is known to regulate gut motility and permeability, secretion of bile acids, bicarbonates, and mucus, as well as mucosal immunity [154], and the microbiota biofilm formation is known to rely on the above gastrointestinal function [155]. The ANS also regulates gut motility, including intestinal transit, which controls the rates of nutrient delivery and bacterial clearance. The migrating motor complex is known as gastric motility in the fasting state, which is controlled by the ANS [156], and impaired migrating motor complex can lead to bacterial overgrowth in the small intestine [157]. In addition, the ANS can impact intestinal barrier integrity via directly altering the permeability of intestinal epithelial cells and modulating the intestinal mucus layer by affecting the mucus secretion of intestinal goblet cells [29,158].

## 5. Microbiota-Gut-Liver Axis

The gut and liver communicate with each other through the portal vein, biliary tract, and systemic circulation. In the intestine, gut microbiota and the host metabolize carbohydrates, lipids, and amino acids from the diet together with endogenous bile acids, and all the metabolites go directly into the liver through portal vein and then affect liver functions. Meanwhile, the liver is linked to the intestine and gut microbiota, as it secretes bile acids and other bioactive substances into the biliary tract and systemic circulation [159]. Thus, microbiota play an important role in metabolic regulation through microbiota-gut-liver axis.

### 5.1. Involvement of Bile Acids in the Microbiota-Gut-Liver Axis

The primary bile acids, such as cholic acid (CA) and chenodeoxycholic (CDCA), are synthesized from cholesterol in the liver, secreted by hepatocytes and then enter the duodenum through the biliary tract. In the intestine, gut microbiota contribute to deconjugation and dehydroxylation of primary bile acids to convert them to secondary bile acids, such as deoxycholic acid (DCA) and lithocholic acid (LCA) [82]. These bile acids aid in the digestion and absorption of dietary fats, cholesterol, and fat-soluble vitamins. Approximately 95% of bile acids are reabsorbed in the terminal ileum and recirculated to the liver through the portal vein, and only a small amount are excreted from the body through feces [160]. This bile acid circulation between the gut and the liver is called “bile acid enterohepatic circulation”.

It has been reported that nuclear receptor FXR and the membrane receptor TGR5, which are involved in the regulation of glucose, lipids and energy balance, are also related to bile acid metabolism. [83]. Bile acids are endogenous ligands of FXR and TGR5, and bile acids activate FXR to help maintain metabolic homeostasis [161]. It has also been shown that TGR5, which is activated by secondary bile acids, such as DCA and LCA, can induce GLP-1 secretion via EECs. GLP-1 can increase insulin secretion in pancreatic β cells and maintain glycemic homeostasis. In addition, FXR has been reported to crosstalk with TGR5 to regulate GLP-1 secretion and maintain metabolic homeostasis [162]. Interestingly, bile acid receptor activation of FXR and TGR5 can relieve metabolic diseases, such as obesity, type 2 diabetes, dyslipidemia, NAFLD, and atherosclerosis [163,164].

Gut microbiota and bile acids interact with and regulate each other. Pathak et al. demonstrated that gut microbiota played an important role in bile acid metabolism and FXR/TGR5/GLP-1 signaling, which promoted adipose tissue browning and improved hepatic glucose and insulin sensitivity [82]. In this study, intestinal FXR agonist fexaramine (FEX) altered the gut microbiota to increase *Acetatifactor* and *Bacteroides*, which converted taurochenodeoxycholic acid (TCDCA) to LCA and then activated TGR5 to induce GLP-1 secretion [82]. Further, it has been shown that gut microbiota contribute to FXR activation via bile acids [165]. Activation of FXR promotes the synthesis of fibroblast growth factor 19 (FGF19) [165], which enters the liver through the portal vein and reduces *de novo* bile acid synthesis by inhibiting CYP7A1 in hepatocytes [166]. Therefore, gut microbiota are involved in regulating bile acid synthesis in the liver. Previous studies have also reported that bile acids can activate FXR to produce antimicrobial peptides, such as human β defensin-1 and 2 (HβD), which inhibit the overgrowth of gut microbiota and help maintain intestinal mucosal barrier function and regulate inflammation [167,168]. In general, gut microbiota can modify the composition and diversity of bile acids [169]. These bile acids will differentially affect the composition and proportion of gut microbiota, depending on their composition [170,171].

### 5.2. Effect of Intestinal Permeability on the Liver

The intestinal barrier helps maintain the structure and function of the intestinal tract and prevents harmful substances, such as bacteria and toxins, from entering other tissues, organs, and blood circulation. The intestinal barrier includes the intestinal mucosa epithelium, intestinal mucus, intestinal microbiota, secreted immunoglobulin, intestinal-associated lymphoid tissue, bile salts, and hormones [172]. When the intestinal barrier is impaired, gut microbiota and other toxins can enter the liver through the portal vein, which can affect liver function and lead to liver damage and various inflammatory and metabolic diseases, such as alcoholic liver disease, NAFLD, primary biliary cholangitis, primary sclerosing cholangitis, and cirrhosis [173].

### 5.3. Systemic Circulation Related to the Microbiota-Gut-Liver Axis

Gut microbiota produce SCFAs, such as formate, acetate, propionate, and butyrate, which are related to maintaining the intestinal epithelium and permeability [11]. SCFAs are mainly metabolized by enterocytes and the liver, and thus play an important role in the microbiota-gut-liver axis. SCFAs are associated with hepatic metabolism of carbohydrates and lipids and help to maintain hepatic energy homeostasis and whole-body energy metabolism [174]. In addition, several kinds of liver diseases are associated with alterations in SCFA levels. For example, mice with alcohol-induced liver injury have shown decreased concentrations of straight-chain SCFAs as compared to normal mice [175]. Further, higher fecal acetate and propionate together with a higher abundance of SCFA-producing gut microbiota were observed in NAFLD patients [176].

Gut microbiota can break down dietary nutrients rich in methylamines, choline, phosphatidylcholine and carnitine to produce trimethylamine (TMA) via the action of TMA lyases. Gut microbiota-derived metabolites, such as TMA, can transfer to the liver through the portal vein. TMA, which is also associated with hepatic lipid metabolism, can be converted into trimethylamine-N-oxide (TMAO) by liver flavin monooxygenase 3 (FMO3) [177,178,179]. It has been shown that TMAO plasma level is correlated with gut microbiota composition [180]. A recent study reported that TMAO down-regulated intestinal and liver cholesterol as well as bile acid metabolism and impaired macrophage reverse cholesterol transport. TMAO can also contribute to dyslipidemia by regulating hepatic lipogenesis and gluconeogenesis [181]. Therefore, increased systemic circulation of TMAO is associated with intestinal dysbiosis, insulin resistance, hypertriglyceridemia, hepatic steatosis, NAFLD, and cancer [182,183,184].

Immunoglobulin A (IgA) can be produced by the liver and intestinal B cells and plasma cells. It is reported to be the most abundant immunoglobulin isoform in the intestinal lumen. IgA is also recognized as the most important antibody for mucosal immunity, which provides the first line of defense against pathogens and harmful substances in the intestinal tract [185]. IgA synthesis in the liver and intestine is dependent on gut microbiota, as IgA is almost non-existent in GF mice [186]. Conversely, IgA contributes to maintaining health by regulating the composition and function of gut microbiota. A previous study showed that IgA promoted symbiosis and intestinal homeostasis by influencing gut microbiota and mucus-associated functional factor [187]. In another study, IgA-deficient mice had persistent *γ-Proteobacteria* colonization in the intestine, which resulted in continuous intestinal inflammation and metabolic imbalance, and thus intestinal injury [188]. Therefore, IgA is also an important regulator in the microbiota-gut-liver axis.

## 6. Conclusions and Perspectives

The gut microbiota play an important role in the metabolism of carbohydrates, lipids, and amino acids, and also contribute to maintaining intestinal permeability and functions. The vagal nerve system acts as the key pathway that communicates between the CNS and ENS. Vagal afferent neurons express receptors for gut peptides, such as CCK, ghrelin, leptin, PYY, GLP-1, and 5-HT, which gut microbiota can regulate to influence nutrient metabolism. Additionally, bile acids, SCFAs, TMAO, and IgA are known as metabolic regulators through a microbiota-gut-liver axis. Interestingly, many studies have reported that changes in gut microbiota are associated with several metabolic diseases, such as obesity, type 2 diabetes, dyslipidemia, NAFLD, and atherosclerosis. Therefore, modulation of gut microbiota and the related microbiota-gut-brain-liver axis may be a potential new strategy for improving metabolic diseases.
